# Supplementary material for: Divorce and adolescent academic achievement: Heterogeneity in the associations by parental education
Source: PLoS One. 2020 Mar 4;15(3):e0229183. doi: 10.1371/journal.pone.0229183 (PMC7055798; doi:10.1371/journal.pone.0229183)
Supplement: S1 Fig — (PDF) [file pone.0229183.s001.pdf]

S1 Figure. Regression coefficient plot of estimates of GPA by parental divorce and equivalized disposable income (n = 7,739)

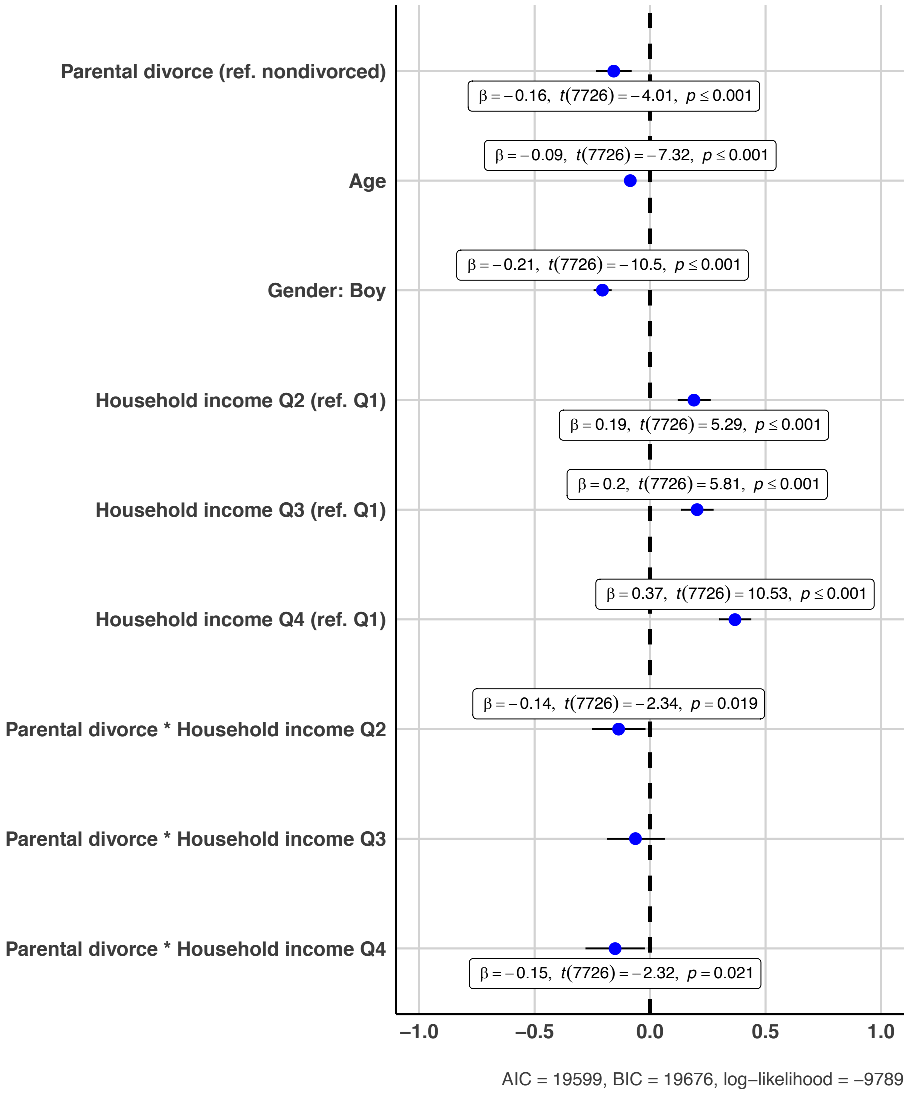

Note. This figure displays a regression coefficient plot whereby the equivalized disposable income was used as an alternative indicator of socioeconomic resources. The blue dots represent the unstandardized beta coefficients (β) while the error bars represent the 95 % confidence intervals. Significant predictors are annotated in the plot with accompanying *t*-statistics and *p*-values. The equivalized disposable income (denoted *Household income* in the figure) was divided into quartiles representing the lowest 25 % to the highest 25%; Q4),
