# Supplementary material for: Divorce and adolescent academic achievement: Heterogeneity in the associations by parental education
Source: PLoS One. 2020 Mar 4;15(3):e0229183. doi: 10.1371/journal.pone.0229183 (PMC7055798; doi:10.1371/journal.pone.0229183)
Supplement: S1 Table — (PDF) [file pone.0229183.s002.pdf]

S1 Table. Comparison of the entire youth@hordaland with adolescents with register-based information

	Entire youth@hordaland	Registry data, current sample
	N = 10257	*N* = 9166
Age (mean (sd))	17.43 (0.84)	17.41 (0.83)
Gender = Boy (%)	4856 (47.3)	4319 (47.1)
Maternal education (%)		
Basic	790 (7.8)	695 (7.7)
Intermediate	3149 (31.3)	2854 (31.6)
High	3699 (36.7)	3355 (37.2)
Unknown	2436 (24.2)	2126 (23.5)
Paternal education (%)		
Basic	799 (8.0)	727 (8.1)
Intermediate	3473 (34.6)	3141 (34.9)
High	3195 (31.8)	2881 (32.0)
Unknown	2583 (25.7)	2259 (25.1)
Economic well-being (%)		
Worse than others	707 (7.1)	633 (7.1)
Equal to others	6696 (67.3)	5999 (67.3)
Better than others	2545 (25.6)	2284 (25.6)

Note. Age and gender stem from register-based information. Information about maternal and paternal educational qualifications, and the adolescents' perception of their family's economic well-being, are self-reported from the youth@hordaland.
