# Supplementary material for: Divorce and adolescent academic achievement: Heterogeneity in the associations by parental education
Source: PLoS One. 2020 Mar 4;15(3):e0229183. doi: 10.1371/journal.pone.0229183 (PMC7055798; doi:10.1371/journal.pone.0229183)
Supplement: Attachment — Submitted filename: Response to Reviewers.docx [file pone.0229183.s004.docx]

**Author Response to Reviewers’ Comments**

Journal:	*PLOS ONE*

Manuscript ID:	PONE-D-19-20535R1

Dear Tomáš Želinský, Ph.D.
Academic Editor, PLOS ONE

Thank you again for allowing us to revise and resubmit our paper. Below are the comments from the reviewers (numbered) and our **responses**. Changes made in the manuscripts are highlighted in *italics*. We have uploaded one version of the manuscript where these changes are identified using track changes (Revised Manuscript with Track Changes”) and one version that is clean (Manuscript).

We thank you for your consideration of our revised manuscript and responses, and we look forward to hearing from you.

Sincerely,

Sondre Aa. Nilsen

(on behalf of all authors)

**Response to Editorial comments:**

1. Once again, I would like to thank you for your patience. Both referees were happy with your adjustments, and they only suggest minor revisions, which I believe, reflects the quality of the improvements you made.

Both referees made a point related to the robustness checks, although from a different perspective. While Reviewer 1 would like to see whether adding "a control measure for time since separation/divorce" could affect your main findings, Reviewer 2 would like to see the results for an additional robustness check using household income as an alternative indicator of a family's socioeconomic resources.

I believe that these suggestions will not affect the main results of your paper, and I will be happy to recommend your paper for acceptance.

**Response:** Thank you for your swift handling of this review process. We have made some additional changes based on the reviewer comments;

- Detailed associations of “time since divorce” on the adolescents’ GPA
- across parental educational levels
- Conducted additional robustness checks using household income
- We have also made some small changes based on reviewer 1 points 1-3, and fixed some typos and grammatical errors that we detected.

The reviewers’ comments and our point-by-point responses can be found below.

**Response to Comments from Reviewer 1**

We thank the reviewer for the efforts of evaluating our manuscript a second time, and for providing constructive means to further improve the paper.

***Reviewer comments:***

1. In the sample section you mention that your analytical sample is similar to the study's full sample in terms of age and gender distribution. It would be helpful to know if the samples are also similar in terms of parental education and income. If the data allow, please mention similarities and differences along other relevant dimensions of the data.

**Response:**

We agree that additional similarities/differences could be useful to add. Unfortunately, we only have register based information about parental income and education on the sample that consented to this register linkage (i.e. the sample that we investigate in this paper). We have, however, compared age, gender, and self-reported parental education and perceived economic well-being between the total sample and the sample consenting to the register linkage. We have uploaded this table as a supplementary file, as we think this information is best suited as a supplement to the paper. As shown in the table the samples were nearly identical across these dimensions between the two samples.

We have updated the sample section which now reads;

*All adolescents born between 1993 and 1995 and residing in Hordaland at the time of the survey were invited (N = 19,439) to participate, and 10,257 agreed, yielding a participation rate of 53 % for the entire study. The present paper is based on a subsample of 9,166 adolescents (47 % of the invited population) who consented to register linkage. This subsample was nearly identical to the total sample with regards to age and gender distribution, and self-reported sociodemographics (see S1 Table).*

2. In the paragraph before the start of the "Statistic Analysis" section, you mention that the divorce group likely includes parents who split up after cohabiting. Please include some estimate from either national or regional data that gives the reader an idea as to what proportion of unions in this study were likely not marriages.

**Response:** Thank you for this suggestion. We agree that additional information about marriage/cohabitation from regional data are useful to the paper. We have added the following based on available regional statistics to the paragraph just before the statistics section;

*Official statistics from Hordaland county in 2012 report that 73.5 % of children and youth below the age of 18 in a two-parent household lived with married parents (the rest with cohabiting parents). Thus, the nondivorced group in the present study most likely contained a group of adolescents with parents that had cohabitated since their birth. As some cohabiting unions eventually marry, we find it likely that the proportion of cohabiting unions in the present sample was somewhat lower than regional estimates also including younger children. Similarly, the divorced group likely contained a group of adolescents whose parents split up from cohabitation. Unfortunately, no official statistics regarding dissolution from cohabiting unions in Norway exists. Our inability to exactly detail the adolescents’ family structure is not unique to the present study but has been rather common within this research field [2]*

3. Your results table presents income measures in units of 1000NOK, while Table 1 describes income in units of 1NOK. You may want to present Table 1 income in units of 1000NOK as well to be consistent with results.

**Response:** Thank you for noticing this inconsistency. We have updated Table 1 so that the units match.

4. In the robustness checks you explore the correlation between parent's education and timing of divorce. You show an average difference of three years in the timing of divorce between the most and least educated parents. This translates into a difference between experiencing divorce around age 5 versus experiencing divorce around age 8. This difference may be important from a developmental framework. It would be worthwhile to control for these differences in your main regressions. Please add update your main models with a control measure for time since separation/divorce. The measure would equal zero for kids who did not experience parental divorce/separation, and it should equal time since event for kids who experienced divorce/separation. This should be a low cost update to models and figures.

**Response:** Thank you for this interesting suggestion. We have spent quite some time debating how adding a measure that equals zero for kids who do not divorce, and time since event for those who do divorce, may affect our models – and especially the interpretations of the models. Firstly, - we have checked this following your advice. The results when adjusting for this variable slightly strengthens all our main findings (i.e., makes the heterogenous effects slightly larger (by the second decimal point)), and do not change the interpretation of the significance level of any of the reported estimates. However, although this might be a mathematically sound method of adding an adjustment to a single level of a dichotomous categorical predictor in a regression (i.e., the “divorced-level” of the “divorce status” variable”), this also appear to permit calculations of impossible/paradoxical values based on the coefficients reported. For instance, the estimates of having non-divorced parents who divorced four years ago.

We therefore prefer to not add this in our main reported analyses as it does not alter the main findings, and may induce some confusions to the interpretations of the estimates. We have, however, conducted further checks of the links between time since divorce, parental educational qualifications, and the adolescents’ GPA. We believe that adding this to the paper is perhaps more illuminating to the question of whether time since divorce is related to the adolescents’ GPA, and whether time since divorce potentially could influence our findings given the slight differences in time since divorce across parental educational qualifications.

We have described this in detail in the statistics section;

*The timing of divorce could potentially covary with parental educational levels and the adolescents’ GPA (e.g., if highly educated parents divorced later on, the estimates of divorce by parental education on the adolescents’ GPA could be influenced by the proximity to the event of dissolution). Moreover, it is possible that associations between timing of divorce and the adolescents’ GPA depend on parental educational qualifications (i.e., that more time spent with highly educated divorced parents differ to time spent with lowly educated divorced parents). We investigate these issues by comparing years since divorce across parental educational levels, and by graphically plotting potential linear and non-linear relationships between timing of divorce and GPA by parental educational qualifications. Generalized additive models (GAMs) were used to investigate potential non-linear relationships. In brief, GAMs may be considered as a semi-parametric extension of the generalized linear model, with the strength of the ability to detect non-linear structures in data that otherwise might be missed [52].*

And added a paragraph to the results-section;

*Adolescents with highly educated parents experienced, on average, that their parents divorced somewhat later (see Figs 3A and 3B). The mean difference in years since divorce among highly educated (i.e., ISCED 7-8) vs. lowly educated (i.e., ISCED 0-2) mothers was about 2.2 years, while the comparable figure among fathers was 2.8 years. Plotting the adolescents’ GPA as a function of years since divorce across the parental educational qualifications (see Fig 4) revealed a slight negative linear association between years since divorce and GPA across most of both maternal and paternal educational levels. The negative association between time since divorce and GPA was strongest among lowly educated mothers. As lowly educated mothers on average had most years since divorce, this finding highlights that time since divorce could not explain the heterogeneity in the associations between divorce and the adolescents’ GPA by maternal educational qualifications. Indeed, the plot suggests that holding years since divorce constant across maternal educational qualifications would slightly strengthen the difference in the negative association between divorce and GPA among adolescents with highly- compared to lowly educated mothers.*

*The plotted GAM curves show some variability around the linear functions for some of the parental educational levels. Overall, these trends do not give any clear indications that GPA is highly influenced by the timing of divorce in the present study.*

And added a paragraph to the discussion;

*Adolescents with highly educated parents experienced, on average, that their parents had divorced somewhat more recently. Time since divorce was overall weakly and negatively associated with the adolescents’ GPA, with some small observable variations across parental educational qualifications. These variations could, however, not explain the heterogeneity found in the present study. Overall, this is generally in keeping with studies that find that youth’s post-divorce adjustment is quite stable or gradually slightly worsen as time passes since the divorce [61,62].*

**Response to Comments from Reviewer 2**

1. Reviewer #2: I thank the authors for taking into account most of my comments and for further improving a already good study. However, I believe that they could conduct one robustness check. In response to my third point, the authors wrote that they do not have information on parental occupation and parental wealth. I see that they cannot then use these variables for a robustness check. However, they can use household income, a variable which is already present in their analysis as a control. I would like to see therefore at least a robustness check using household income as an alernative indicator of a family's socioeocnomic resources than parental education. It is precisely one shortocoming of the previous literature that they only used parental education and this study has the potential to address this shortcoming.

**Response:** We thank the reviewer for assessing our paper a second time, and for the positive comments and encouragements.

We have followed the reviewer’s advice and added additional checks utilizing household income as an alternative indicator of socioeconomic status. Utilizing the equivalized disposable income measure divided into quartiles, we find a similar but weaker pattern than in the models with parental education. We have added a figure summarizing the regression results and uploaded it as a supplementary figure.

We have updated the statistics section;

*Lastly, we performed checks utilizing the income measures as alternative indicators of the family’s socioeconomic resources. The income measures were divided into quartiles (i.e., into four equal parts representing the lowest 25 % to the highest 25%), and the adolescents’ GPA was regressed on the interaction term between parental divorce and the income quartiles (similarly to the procedure described above).*

And made changes to the result section;

*Lastly, using equivalized disposable income (EDI) as an alternative indicator of socioeconomic resources, we found a similar but weaker pattern whereby the negative association between divorce and GPA was relatively stronger among adolescents in the second income quartile (Q_2_; b = - 0.16, p < 0.01) and in the fourth quartile (Q_4_; b = -0.15, p = 0.02) compared to those in the first quartile (Q_1_). The difference between Q_1_ and Q_3_ was not statistically significant (see S1 Fig. with further test statistics). Adjusting the analyses for parental educational qualifications attenuated and removed the significant difference between Q_1_ and Q_4_. No heterogeneity in the associations between divorce and GPA by mothers’ or fathers’ net income were found (results not shown).*

*Of note, it is highly likely that potential heterogeneity by measures of household income in the links between divorce and academic outcomes is sensitive to how income is operationalized. As parental education was the main focus of interest in the present study, we did not examine this any further in the present paper (e.g., other ways of dividing income into categories).*
